# Never in Mitosis Gene A Related Kinase-6 Attenuates Pressure Overload-Induced Activation of the Protein Kinase B Pathway and Cardiac Hypertrophy

**DOI:** 10.1371/journal.pone.0096095

**Published:** 2014-04-24

**Authors:** Zhouyan Bian, Haihan Liao, Yan Zhang, Qingqing Wu, Heng Zhou, Zheng Yang, Jinrong Fu, Teng Wang, Ling Yan, Difei Shen, Hongliang Li, Qizhu Tang

**Affiliations:** 1 Department of Cardiology, Renmin Hospital of Wuhan University, Wuhan, Hubei Province, P. R. China; 2 Cardiovascular Research Institute of Wuhan University, Wuhan, Hubei Province, P. R. China; Northwestern University, United States of America

## Abstract

Cardiac hypertrophy appears to be a specialized form of cellular growth that involves the proliferation control and cell cycle regulation. NIMA (never in mitosis, gene A)-related kinase-6 (Nek6) is a cell cycle regulatory gene that could induce centriole duplication, and control cell proliferation and survival. However, the exact effect of Nek6 on cardiac hypertrophy has not yet been reported. In the present study, the loss- and gain-of-function experiments were performed in Nek6 gene-deficient (Nek6^−/−^) mice and Nek6 overexpressing H9c2 cells to clarify whether Nek6 which promotes the cell cycle also mediates cardiac hypertrophy. Cardiac hypertrophy was induced by transthoracic aorta constriction (TAC) and then evaluated by echocardiography, pathological and molecular analyses *in vivo*. We got novel findings that the absence of Nek6 promoted cardiac hypertrophy, fibrosis and cardiac dysfunction, which were accompanied by a significant activation of the protein kinase B (Akt) signaling in an experimental model of TAC. Consistent with this, the overexpression of Nek6 prevented hypertrophy in H9c2 cells induced by angiotonin II and inhibited Akt signaling *in vitro*. In conclusion, our results demonstrate that the cell cycle regulatory gene Nek6 is also a critical signaling molecule that helps prevent cardiac hypertrophy and inhibits the Akt signaling pathway.

## Introduction

Cardiac hypertrophy is an adaptive response to increased pressure or volume overload to maintain cardiac function. However, prolonged cardiac hypertrophy is a risk factor for arrhythmias, sudden death and heart failure, which represents a major cause of morbidity and mortality [1], [2]. Alterations in signaling transduction pathways and transcription factors that are induced by hypertrophic signals result in cardiac hypertrophy, which is characterized by an increase in cardiomyocyte size, enhanced protein synthesis, altered hypertrophic gene expression, and cell cycle regulation [2], [3]. Cell cycle progression is coupled with the accumulation of cell mass to ensure that cell size is constant after proliferation,but cell growth can also become hypertrophic growth that is uncoupled from proliferation in many diseases. Cardiac hypertrophy appears to be a specialized form of cellular growth that involves the control of proliferation and cell cycle regulation [4]. Nevertheless, the correlation between cardiac hypertrophy and cell cycle progression, and whether the same factors that regulate hyperplastic growth also mediate hypertrophic growth in adult myocytes has been largely ignored [3]. Therefore, it will provide novel molecular targets for prevent cardiac hypertrophy and heart failure to clarify whether the gene responsible for its ability to promote cell cycle also plays a role in regulating cardiac hypertrophy.

NIMA (never in mitosis, gene A)-related kinase-6 (Nek6) is a serine/threonine kinase structurally related to the Aspergillus nidulans protein NIMA, which is essential for the initiation of mitosis [5]. Nek6, as a cell cycle regulatory gene, could induce centriole duplication and regulate cell proliferation and survival [6], [7]. A reduction in the activity of Nek6 has been shown to arrest cells in mitosis [8]. Nassirpour, R., et al. suggested that Nek6 plays a pivotal role in tumorigenesis [9]. Similarly, Salem et al. also found that the deregulation of Nek7, the close paralog of Nek6, could induce oncogenesis [8]. Furthermore, the mechanisms by which Nek6 regulates carcinogenesis have been revealed gradually. An investigation by Vaz Meirelles et al. provided new insights in how hNek6 might be involved in novel signaling pathways and regulate pathways such as the actin cytoskeleton regulation, the cell cycle regulation, Notch signaling, and NF-κB signaling [10]. Lizcano et al. demonstrated that Nek6 phosphorylates S6K1 and SGK1 in vitro [11]. Kang et al. also revealed that Oct1 is phosphorylated at S335 in the Oct1 DNA binding domain by Nek6 [12]. In addition, the overexpression of Nek6 in cells inhibited p53-induced increases in the intracellular levels of reactive oxygen species (ROS) [13]. These studies suggest that Nek6 plays a pivotal role in cell cycle regulation and the phosphorylation of signaling pathways. However, the exact effect of Nek6 on cardiac hypertrophy has not yet been reported. Therefore, we for the first time used *Nek6*-deficient mice to clarify whether the Nek6 is responsible for regulating cell cycle as well as cardiac hypertrophy. Interestingly, our data demonstrated that Nek6 attenuated pressure overload-induced activation of the protein kinase B (Akt) pathway and cardiac hypertrophy.

## Materials and Methods

### Ethics statement

The study was approved by the Renmin Hospital of Wuhan University Human Research Ethics Committee. All the samples were collected after written informed consent. All animal studies were performed in accordance with the guidelines of the NIH (Guide for the Care and Use of Laboratory Animals, 1996), and were approved by the Animal Care and Use Committee of Renmin Hospital of Wuhan University.

### Human heart samples

The left ventricular samples were obtained from dilated cardiomyopathy (DCM) explanted patients during heart transplantation. The control samples were obtained from donors with normal cardiac function.

### Animal models

Nek6 knockout (KO) mice and their wild-type littermates (male, aged 8–10 weeks, body weight of 24–27 g) were subjected to sham or transthoracic aorta constriction (TAC) operations. The source of the Nek6^−/−^ mice was the European Mouse Mutant Archive (EMMA: 02372). TAC was performed as described previously [14]. Mice were anaesthetized and a horizontal skin incision was made at the 2–3 intercostal space. The descending aorta was isolated and a blunt 26-gauge needle was placed next to the aorta, and a 7–0 silk suture was then tied around the needle and the aorta. The needle was quickly removed after ligation. The mice of sham groups underwent the same procedure, but without ligation. The hearts and lungs were harvested and weighed, and the tibial lengths were measured to compare the heart weight/body weight (HW/BW, mg/g), lung weight/body weight (LW/BW, mg/g), and heart weight/tibial length (HW/TL, mg/cm) ratios among the different groups.

### Materials

The anti-Nek6 (ab76071) antibody against both human and mice was purchased from Abcam. The primary antibodies against phosphor (P)-Akt^Thr308^ (2965), total (T)-Akt (4691), P -GSK-3β^Ser9^ (9322), T-GSK-3β (9315), P-mTOR^Ser2448^ (2971), T-mTOR (2983), P-4EBP1 (2855p), T-4EBP1 (9644p), P-eIF4e (9741), T-eIF4e (2067) and GAPDH (2118) were purchased from Cell Signaling Technology. The goat anti-rabbit secondary antibodies (LI-COR, 926-32211) were used for Western blotting. The BCA protein assay kit was obtained from Thermo Scientific (23225), and all other reagents were purchased from Sigma.

### Echocardiography

Echocardiography was performed using a MyLab30CV ultrasound instrument equipped with a 10-MHz linear array ultrasound transducer (Biosound ESAOTE Inc.) to assess the wall thickness and internal diameter of the left ventricle (LV). The LV end-systolic and end-diastolic diameters (LVESD, LVEDD) were measured from the M-mode tracing at the level of the mid-papillary muscle with a sweep speed of 50 mm/s [15]. The mean values were obtained from three different cardiac cycles for each assessment.

### Histological analysis and immunohistochemistry

Hearts were excised, weighed, fixed with 10% paraformaldehyde for 12–24 h, and embedded in paraffin. The paraffin sections were cut transversely into 5-µm-thick sections, deparaffinized, and subsequently stained with hematoxylin and eosin (H&E) to determine the myocyte cross-sectional area (CSA). Fibrosis was assessed using Picro-Sirius red (PSR) staining to determine collagen deposition. Fibrillar collagen was identified through its red appearance in the sections. Individual myocytes were measured and the sections were analyzed morphometrically using Image Pro-Plus version 6.0 image analysis software. The expression of Nek6 after TAC was detected in the mouse heart sections using immunohistochemical staining with anti-Nek6 antibody, and the nuclei were counterstained with hematoxylin. Immunofluorescence staining with anti-α-actinin and DAPI was performed in the H9c2 cells to observe the myocyte hypertrophy.

### Cell culture

The H9c2 cell line derived from rat embryonic heart tissue were cultured as described previously with a minor revision for Nek6 transfection to assess the effect of Nek6 overexpression *in vitro* [16]. H9c2 cells were seeded onto six-well culture plate at a density of 1×10^6^cells/well, and transfected with the pCMV-SPORT6-Nek6 vector (Open Biosystem, Clone ID: 4209097) after serum starved for 24 hours. Subsequently, cells were stimulated with angiotonin II (Ang II, 1 µM) and harvested for protein extraction at the indicated times. For surface area measurements, the cells were fixed, permeabilized in 0.1% Triton X-100, and stained with anti-α-actinin (Millipore, 05–384, 1∶100) followed by Alexa FluorH 488-conjugated goat anti-mouse IgG secondary antibody (Invitrogen, A11004). The myocytes were mounted in anti-fade reagent with DAPI (Invitrogen, S36939).

### Western blotting and real-time quantitative PCR

The protein extracts were harvested from different groups of cardiac tissues or cardiomyocytes that had been lysed in RIPA buffer. After measuring protein concentration with a BCA protein assay kit, equal amounts of protein were resolved by polyacrylamide gel electrophoresis and transferred onto polyvinylidene difluoride membranes (Millipore, Cat. No. IPFL00010). The membranes were then probed with specific primary antibodies, and incubated with secondary antibody (LI-COR, 926-32211). The blots were scanned and visualized using an Odyssey Infrared Imaging System (Odyssey, LI-COR). The expression levels of specific proteins were normalized to GAPDH levels on the same membrane.

The total RNA was extracted by TRIzol reagent (Roche, 7950567275) to detect the mRNA levels of hypertrophic and fibrotic markers. After measuring the concentration and purity of RNA with a SmartSpec Plus Spectrophotometer (Bio-Rad), the RNA was reverse-transcribed into cDNA using a Transcriptor First Strand cDNA Synthesis Kit (Roche, 04896866001). PCR amplifications were performed using the LightCycler 480 SYBR Green I Master kit (Roche, 04887352001) according to the manufacturer's instructions. Each sample was run in triplicate. and the mRNA levels of the gene were normalized to GAPDH expression.

### Statistical analysis

All of the data are presented as the mean ±S.E.M. Data were analyzed using ANOVA followed by LSD or Tamhane's test, using SPSS17.0 software. A *P*-value <0.05 was considered to be statistically significant.

## Results

### Nek6 expression in human failing hearts and experimental hypertrophic models

**Figure 1:**
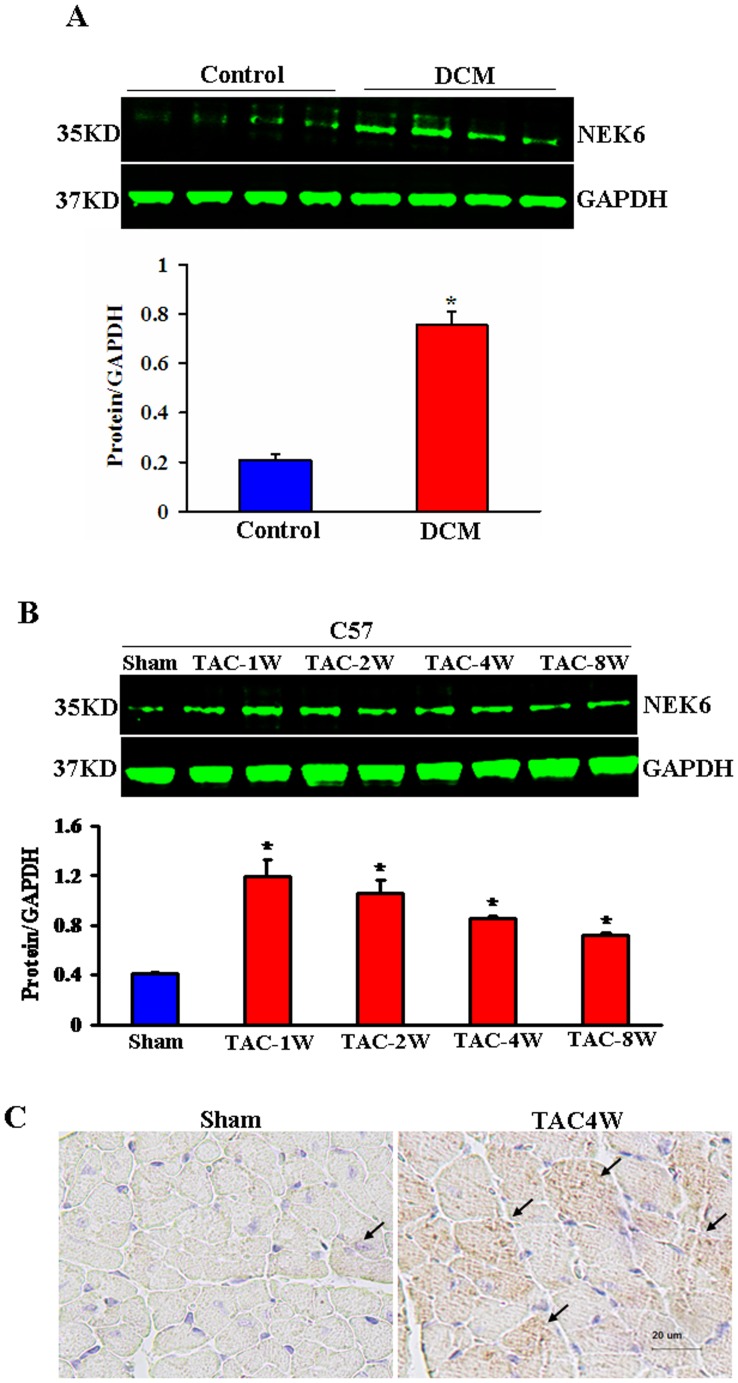
Nek6 expression in human failing hearts and experimental hypertrophic models. A, The protein levels of Nek6 in donor hearts and human failing hearts from DCM patients (n = 4). Top, representative western blots; Bottom, quantitative results. *P<0.05 *vs*. control donor hearts. B, The protein levels of Nek6 in WT hearts after TAC at the indicated time points. Top, representative western blots; Bottom, quantitative results. *P<0.05 *vs*. sham. C, Immunostaining for cardiac Nek6 protein expression in WT hearts after TAC or sham operations. The brown area indicated by the arrows shows the location of Nek6-positive staining.

We first detected the Nek6 expression in LV myocardium samples from DCM patients undergoing heart transplants and donors by western blotting. Nek6 was upregulated in the failing hearts of the DCM patients compared with normal donors (Figure 1A). We further examined Nek6 levels in the heart tissues of wild-type (WT) mice that underwent experimental cardiac hypertrophy models induced by TAC in different durations. It was novel to find that the expression of Nek6 was increased significantly after TAC, but then decreased gradually from 2 weeks after TAC (Figure 1B). This suggests that a compensatory increase and subsequent de-compensatory decrease in Nek6 expression occurs during the progress of the cardiac hypertrophy. Moreover, the immunostaining was performed to identify the localization of Nek6. The results of immunohistochemistry demonstrated that the expression of Nek6 in the cardiomyocytes of wild-type mice subjected to TAC was increased obviously compared with the sham-treated mice (Figure 1C). These data suggest that Nek6 is expressed in the cardiomyocytes, and might be involved in the progress of cardiac hypertrophy.

### Cardiac hypertrophy is aggravated in Nek6-deficient mice after TAC

**Figure 2:**
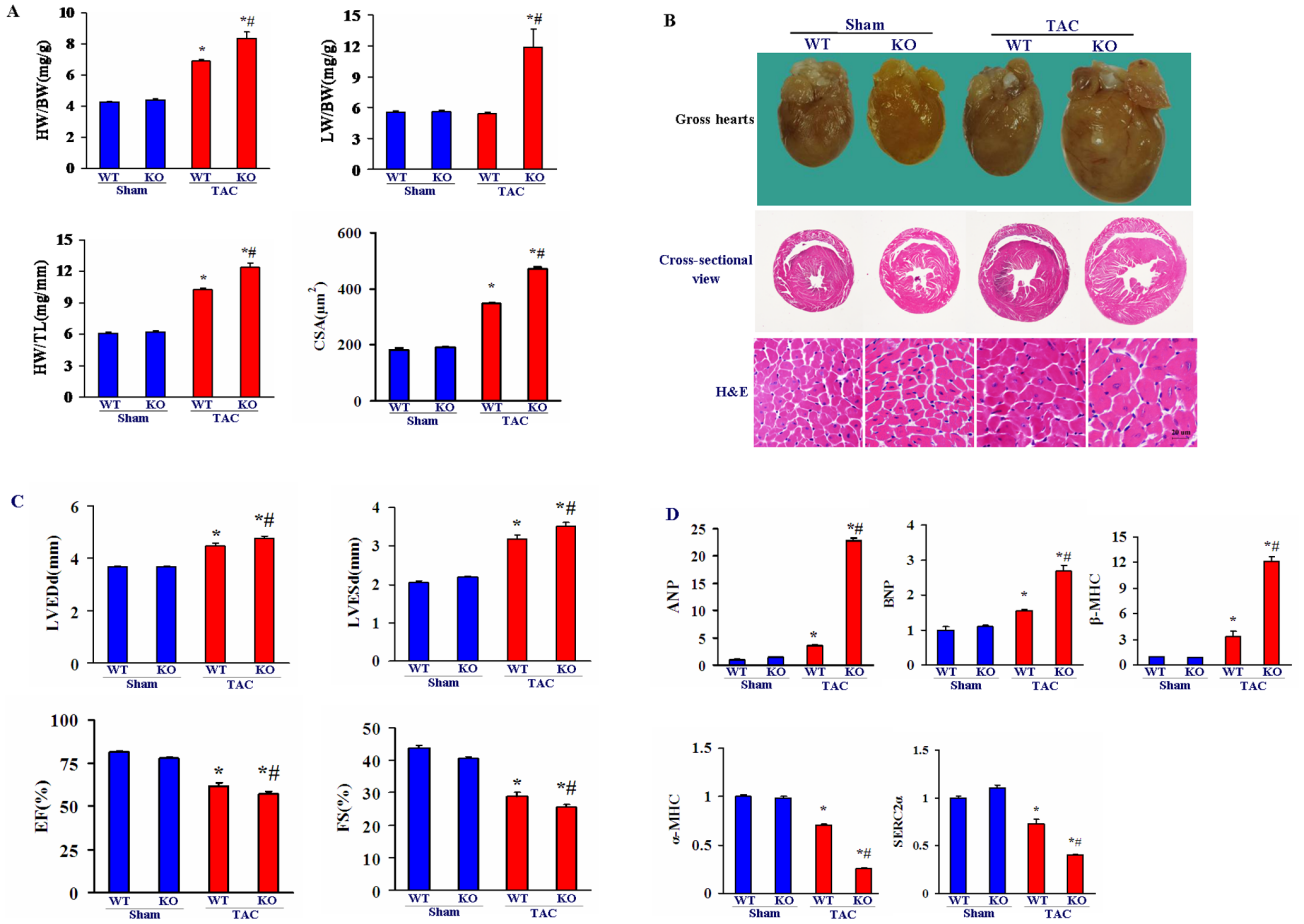
Cardiac hypertrophy is aggravated in Nek6-deficient mice after TAC. A, Statistical results of the HW/BW, LW/BW, HW/TL ratios, and myocyte cross-sectional areas of WT and Nek6 KO mice 4 weeks after TAC or sham surgery. B, Gross hearts, a cross-sectional view of the whole hearts, and H&E staining of the indicated groups (scale bars  = 20 mm). C, Echocardiography measurements of the indicated groups. D, The mRNA levels of the hypertrophic markers ANP, BNP, β-MHC, α-MHC, and SERCA2α detected by real-time quantitative PCR in the indicated groups. *P<0.05 *vs*. WT/sham; # *P*<0.05 *vs*. WT/TAC.

To further elucidate the role of Nek6 in the development of cardiac hypertrophy, Nek6 ablation (Nek6^−/−^) mice were used to perform the loss-of-function experiments. Under basal fed conditions, Nek6^−/−^ mice did not show marked alterations in phenotype compared with WT mice. Four weeks after TAC surgery, HW was increased in both Nek6^−/−^ and WT mice, and the increase in HW was more pronounced in Nek6^−/−^ mice than WT mice, whether expressed as HW/BW or HW/TL. The TAC-induced increase in the LW/BW was also higher in the Nek6^−/−^ mice compared with WT controls (Figure 2A). Histological analyses of gross heart and H&E-stained LV tissue sections revealed an enhanced effect of Nek6 deficiency on pressure overload-induced cardiac hypertrophy (Figure 2B). TAC also significantly increased the cardiomyocyte cross-sectional area (CSA) in both WT and Nek6^−/−^ mice, but the increase was greater in Nek6^−/−^ mice than WT controls after TAC (Figure 2A). No differences in heart rate or blood pressure were observed under any experimental conditions.

Next, cardiac hypertrophy and left ventricular function were evaluated by echocardiography. TAC led to a significant increase in both LVESD and LVEDD, but the increase was significantly higher in Nek6^−/−^ mice compared with wild-type 4 weeks after surgery. The LV ejection fraction (EF) and fractional shortening (FS) were significantly decreased in Nek6^−/−^ mice 4 weeks after TAC, which demonstrated the aggravation of LV function in Nek6^−/−^ mice compared with WT controls (Figure 2C).

In addition, the mRNA levels of hypertrophic markers were detected by real-time quantitative PCR. The expression of atrial natriuretic peptide (ANP), B-type natriuretic peptide (BNP), β-myosin heavy chain (β-MHC) and α-myosin heavy chain (α-MHC), and sarcoendoplasmic reticulum Ca^2+^-ATPase (SERCA2α) were similar in the WT and Nek6^−/−^ mice under sham conditions, but were markedly changed after TAC. Moreover, the upregulation of ANP, BNP, and β-MHC, and the downregulation of α-MHC, and SERCA2α were more prominent in Nek6^−/−^ mice than WT 4 weeks after TAC (Figure 2D).

### Cardiac fibrosis is augmented in Nek6 deficient mice after TAC

**Figure 3:**
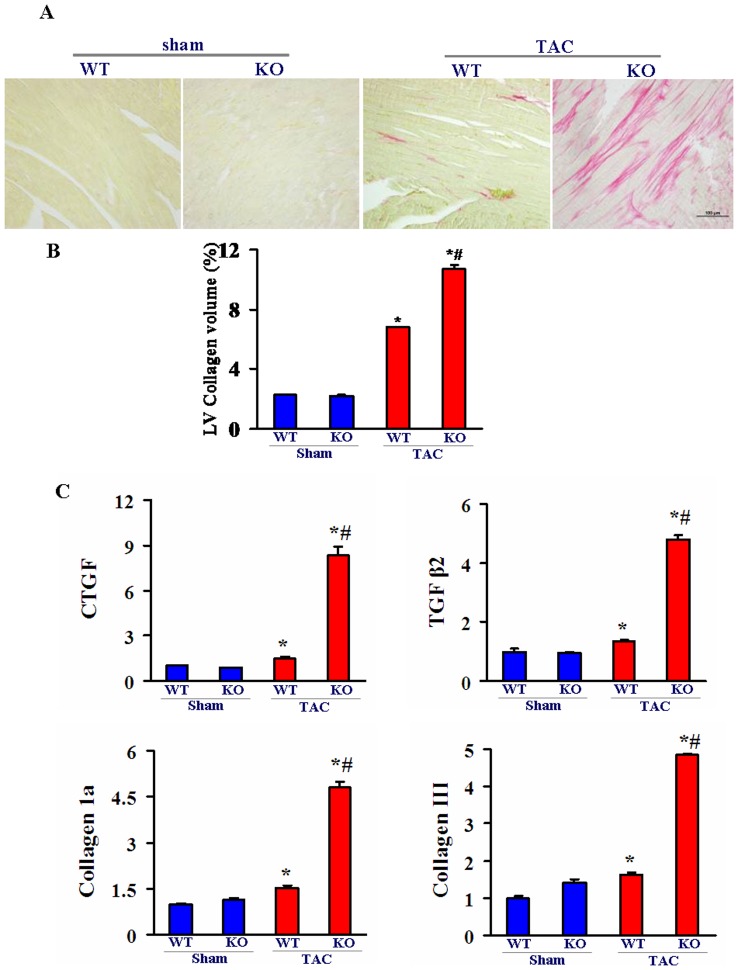
Cardiac fibrosis is augmented in Nek6-deficient mice after TAC. A, PSR staining of the left ventricular sections was performed in the WT and Nek6 KO mice 4 weeks after TAC or sham surgery (scale bar  = 20 µm). B, The fibrotic area of the histological sections was quantified by an image analysis system. C, The mRNA levels of the fibrosis markers CTGF, TGF-β2, collagen I and collagen III in the myocardium were detected by real-time quantitative PCR in the indicated groups. *P<0.05 *vs*. WT/sham; # *P*<0.05 *vs*. WT/TAC.

Cardiac hypertrophy is always associated with increased fibrosis of the interstitium. We therefore performed PSR staining and detected the expression of fibrosis markers to assess fibrosis. The collagen deposition in the myocardial interstitium was augmented in both WT and Nek6^−/−^ mice 4 weeks after TAC, but the degree of fibrosis was much more prominent in the Nek6^−/−^ mice (Figure 3A). Quantitative analysis of the LV collagen volume also revealed substantially increased fibrosis in the Nek6^−/−^ mice, which is consistent with the results of the PSR staining (Figure 3B). In addition, the mRNA levels of fibrosis markers were detected by real-time quantitative PCR. The expression of connective tissue growth factor (CTGF), transforming growth factor (TGF)-β2, collagen I, and collagen III were similar in WT and Nek6^−/−^ mice under sham conditions, but were markedly increased in both WT and Nek6^−/−^ mice after TAC. Moreover, the upregulation of CTGF, TGF-β2, collagen I, and collagen III were more prominent in the Nek6^−/−^ mice than WT mice 4 weeks after TAC (Figure 3C).

### Nek6 overexpression attenuates Ang II-induced myocyte hypertrophy *in vitro*

**Figure 4:**
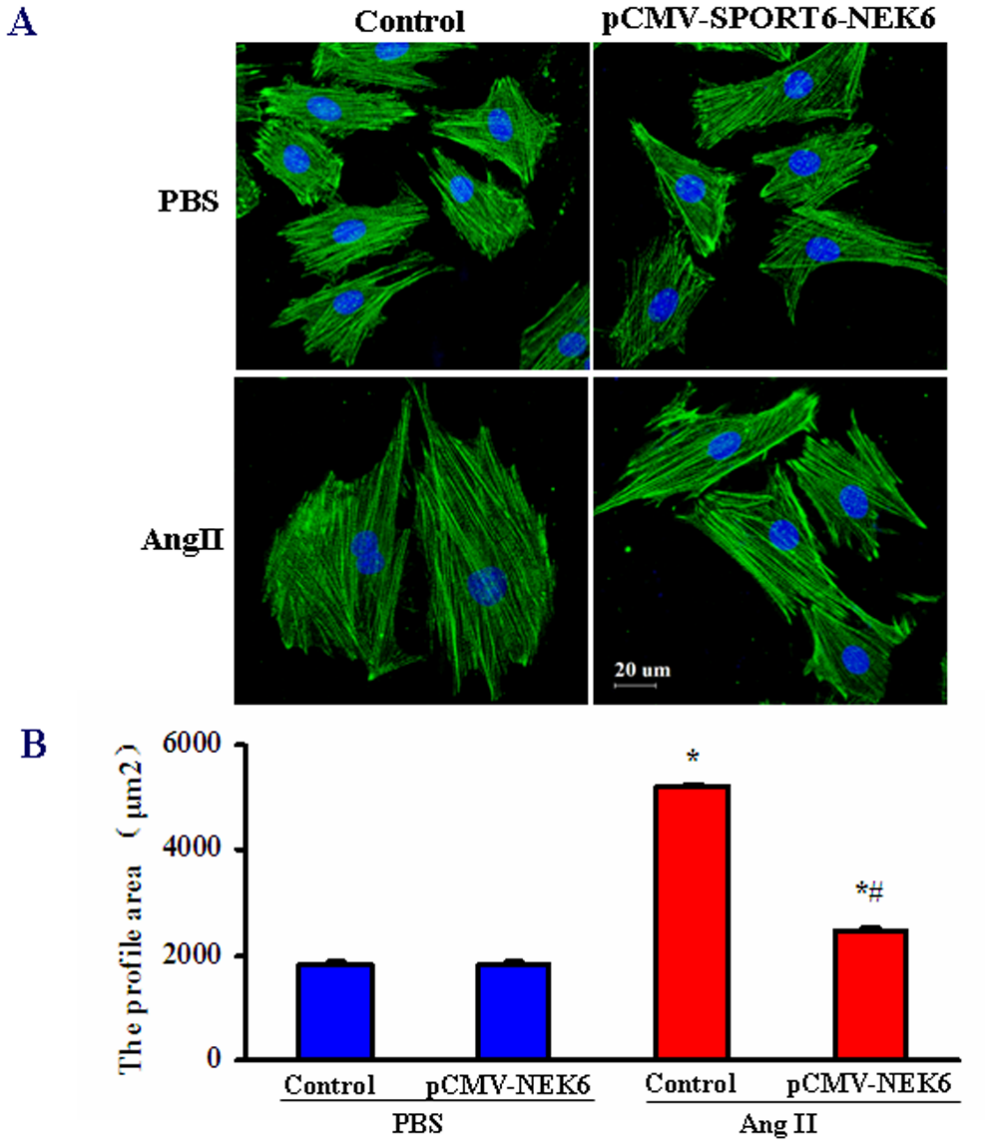
Nek6 overexpression attenuates Ang II-induced myocyte hypertrophy. A, Representative images of the cardiomyocytes revealed the inhibitory effect of Nek6 on the enlargement of cardiomyocytes in response to 24(scale bar  = 20 µm). B, Quantification of the profile area by measuring 100 random cells. *P<0.05 *vs*. control/PBS group; # *P*<0.05 *vs*. control/Ang II group.

In order to further establish the effects of Nek6, we overexpressed Nek6 in cultured H9c2 rat cardiomyocytes to perform gain-of-function studies. The cells were transfected with pCMV-SPORT6-Nek6 or empty vector, and were then treated with Ang II for the indicated times. Staining with anti-α-actinin and DAPI was then performed to evaluate the profile area of cardiac myocytes. Ang II treatment increased the profile area of H9c2 cells, an indication of hypertrophy, but the overexpression of Nek6 significantly attenuated the Ang II-induced cell enlargement (Figure 4A and B). These data suggest that Nek6 protected cardiomyocytes from hypertrophy *in vitro*.

### The effect of Nek6 on Akt/GSK-3β signaling *in vivo* and *in vitro*

**Figure 5:**
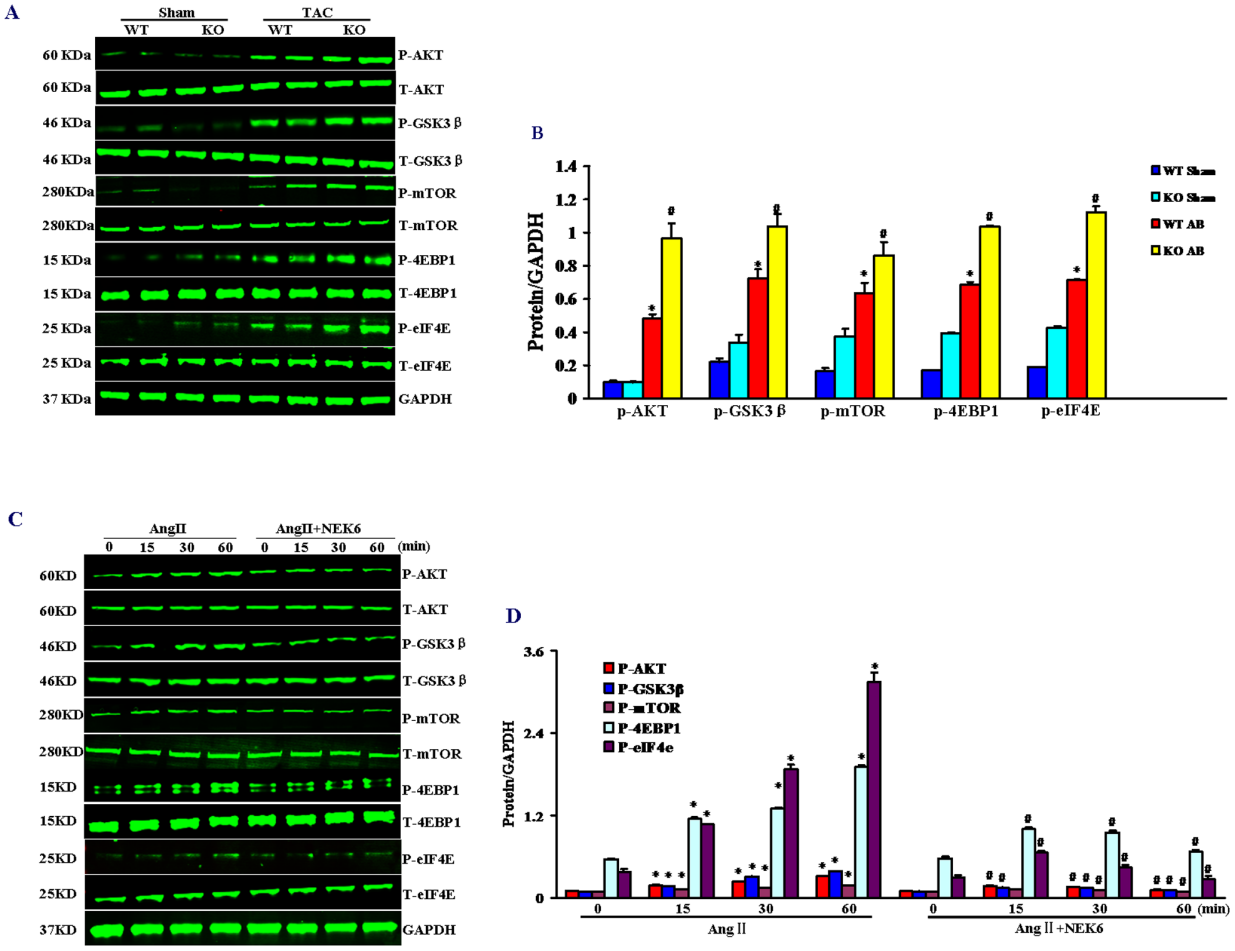
The effect of Nek6 on Akt signaling *in vivo* and *in vitro*. A and B, The phosphorylated and total protein levels of Akt, GSK-3β, mTOR, eIF4E, and 4E-BP1 in WT and Nek6 KO mice 4 weeks after TAC or sham surgery. Nek6 deficiency augments the activation of Akt signaling. A, representative western blots (duplicate lanes represent two different heart samples); B, quantitative results. *P<0.05 *vs*. WT/sham; # *P*<0.05 *vs*. WT/TAC. C and D, phosphorylated and total protein expression levels of Akt, GSK-3β, mTOR, eIF4E, and 4E-BP1 in H9c2 cells treated with Ang II for the indicated times with or without (control) the overexpression of Nek6. Nek6 overexpression attenuated the activation of Akt signaling. C, representative western blots; D, quantitative results. *P<0.05 vs. the control group at the 0 time point; # *P*<0.05 *vs*. the control group at the same time point.

We and others previously revealed that several signaling pathways are involved in the regulation of cardiac hypertrophy, including the MAPK, calcineurin/nuclear factor of activated T cells (NFAT), and Akt/GSK-3β signaling cascades [14], [15], [17]. Nek6 is considered as a cell cycle regulatory gene, we therefore investigated the effects of Nek6 on Akt/GSK-3β signaling, which also plays a role in regulating the cell cycle, to explore the molecular mechanisms by which Nek6 regulates the hypertrophic response. Western blotting results blotting that Nek6 deficiency markedly enhanced the phosphorylation level of Akt and GSK-3β compared with WT controls after TAC (Figure 5A and B). This was accompanied by augmented Akt and GSK-3β phosphorylation. The phosphorylation levels of mammalian target of rapamycin (mTOR), eukaryotic initiation factor 4E (eIF4E), and eIF4E-binding protein 1(4E-BP1) were also increased in Nek6^−/−^ mice compared with WT controls after TAC (Figure 5A and B). Therefore, Nek6 deficiency promoted the activation of Akt/GSK-3β signaling *in vivo*.

To further confirm the effect of Nek6 on Akt/GSK-3β signaling, the gain-of-function studies were performed. Nek6 overexpressed H9c2 cardiomyocytes were treated with Ang II for the indicated times, and the phosphorylation levels of Akt and GSK-3β were assessed *in vitro*. As shown in Figures 5C and D, the phosphorylation levels of Akt, GSK-3β, mTOR, eIF4E, and 4E-BP1 were decreased significantly in Nek6 overexpressed H9c2 cardiomyocytes compared with control H9c2 cells after AngII-stimulation at the same time point. Therefore, Nek6 overexpression inhibited Akt/GSK-3β signaling *in vitro*.

## Discussion

In the present study, we got novel findings that the expression of Nek6 was upregulated in human failing hearts, and was markedly induced in experimental hypertrophic models. We further demonstrated that the absence of Nek6 promoted cardiac hypertrophy, fibrosis, dilatation and cardiac dysfunction, which was accompanied by the significant activation of Akt/GSK-3β signaling in an experimental model of TAC. Conversely, the overexpression of Nek6 prevented Ang II-induced hypertrophy in H9c2 cardiomyocytes and inhibited Akt/GSK-3β signaling *in vitro*.

This study established that Nek6 is involved in the development of cardiac hypertrophy both *in vivo* and in *vitro*. Cardiac hypertrophy characterized by increase in cardiomyocyte size is a risk factor for heart failure and sudden death [18]. Previous studies revealed that the cardiomyocyte apoptosis and autophagy, interstitial fibrosis, angiogenesis, metabolic disorders, and fetal gene expression were involved in pathological cardiac hypertrophy [19]. Recently, increasing data proposed that cardiac hypertrophy appears to be a specialized form of cellular growth that involved proliferation control and cell cycle regulation [4]. The proteins classically thought to be involved in cell cycle regulation also play a critical role in the controlling of cellular growth [3]. For instance, it was reported that CycD/Cdk4 and Cdk9 were implicated in regulating cardiac hypertrophy in mammalian cells [20], [21], [22], and Myc is required for a normal hypertrophic response [23]. CycD/Cdk4 and Myc were considered as factors implicated in regulating both cell size and number [3], [24], [25]. Nek6 is also a cell cycle regulatory gene whose function is important for mitotic progression [7], [26]. It has been demonstrated that hNek6 transcripts are ubiquitously expressed, and the highest expression is found in the heart and skeletal muscle, as determined by northern blotting [27]. In the current study, we observed that the expression of Nek6 was increased both in human failing hearts and experimental models of hypertrophy by western blotting and immunostaining. We deduced that the increased Nek6 might be a compensatory response of the heart to rivalry pressure overload-induced cardiac hypertrophy. The loss- and gain-of-function experiments were performed in Nek6^−/−^ mice and H9c2 cells transfected with the vector of pCMV-SPORT6-Nek6 to further elucidate the role of Nek6 in the development of cardiac hypertrophy. Nek6^−/−^ mice displayed augmented cardiac hypertrophy, dilatation, fibrosis and aggravated cardiac dysfunction, and the overexpression of Nek6 in H9c2 cells also confirmed the inhibitory effects of Nek6 on hypertrophy of cardiomyocytes. Taken together, the above results strongly suggest that Nek6 plays an important role in preventing the pathological processes of cardiac hypertrophy.

We further found that Nek6 was implicated in the regulation of the Akt/GSK-3β signaling during the progress of cardiac hypertrophy both *in vivo* and in *vitro*. Previously studies have demonstrated that a number of signaling pathways including the MAPK, Akt/GSK-3β and calcineurin/nuclear factor of activated T cells (NFAT) signaling cascades play roles in cardiac hypertrophy [14], [15], [17]. The Akt/GSK-3β signaling pathway has been implicated in multiple cellular processes, including migration, proliferation and regulation the progression of cell cycle [28], [29]. Kida et al also reported that Akt activation mediates cell-cycle progression by phosphorylating and consequently inhibiting GSK-3β [30], [31]. Taking into account the critical role of Akt/GSK-3β in regulation the progress of both cardiac hypertrophy and cell cycle, we next examined the activation of this signaling pathway. We observed that the phosphorylation level of Akt and GSK-3β was markedly enhanced in Nek6^−/−^ mice, and significantly decreased in the Nek6 overexpressed H9c2 cardiomyocytes. What's more, we got a novel finding that the phosphorylation level of mTOR, another serine/threonine kinase downstream of Akt, was markedly increased in Nek6^−/−^ mice. The Akt/mTOR signaling pathway has also been found to be a key signaling cascade that regulates the cell cycle, proliferation and hypertrophy [32], [33], and the mTOR pathway mediates the phosphorylation of the ribosomal protein S6 kinases and eukaryotic translation initiation factor 4E binding protein 1 leading to the release of the translation initiation factor eIF4E [34]. Consistent with previous studies, we also found that the phosphorylation levels of 4E-BP1 and eIF4E were increased in Nek6^−/−^ mice, but that there was no difference in p70S6k phosphorylation between Nek6^−/−^ and WT mice after TAC. The corresponding decrease in mTOR, 4E-BP1 and eIF4E phosphorylation levels was observed in Nek6 overexpressing H9c2 cardiomyocytes compared with control H9c2 cells after Ang II-stimulation. Combining the results of previous reports with the current study, we speculate that the Akt signaling pathway at least partially contributes to the inhibitory effects of Nek6 on cardiac hypertrophy. However, further investigations are needed to establish how Nek6 regulates the Akt signaling pathway.

In summary, our present work provides *in vivo* and *in vitro* evidence that the expression of Nek6 prevents the cardiac hypertrophy, possibly due to block Akt signaling pathway activities that are unrelated to cell cycle progression. These data support a novel finding that the cell cycle regulatory factor Nek6 is also a critical signaling molecule that plays role in the development of cardiac hypertrophy. Therefore, Nek6 could be another new effective therapeutic target against cardiac hypertrophy and heart failure. The present study might shed light on the pathogenesis and molecular mechanisms of cardiac hypertrophy, as well as provide novel strategies for the treatment of cardiac hypertrophy.
